# Deletion of *Limk1* and *Limk2* in mice does not alter cochlear development or auditory function

**DOI:** 10.1038/s41598-019-39769-z

**Published:** 2019-03-04

**Authors:** Qiaojun Fang, Yuhua Zhang, Peng Da, Buwei Shao, Haolai Pan, Zuhong He, Cheng Cheng, Dan Li, Jiaqi Guo, Xiaohan Wu, Ming Guan, Menghui Liao, Yuan Zhang, Suhua Sha, Zikai Zhou, Jian Wang, Tian Wang, Kaiming Su, Renjie Chai, Fangyi Chen

**Affiliations:** 1 Department of Biomedical Engineering, Southern University of Science and Technology, 518000 Shenzhen, China; 2 Key Laboratory for Developmental Genes and Human Disease, Ministry of Education, Institute of Life Sciences, Southeast University, 210096 Nanjing, China; 3 Department of Otolaryngology-Head and Neck Surgery, Affiliated Hospital of Nantong University, 226001 Nantong, China; 4 Department of Otolaryngology, Affiliated Sixth People’s Hospital, Shanghai Jiao Tong University, 600 Yishan Road, 200233 Shanghai, China; 5 Department of Pathology and Laboratory Medicine, Medical University of South Carolina, 29425 Charleston, South Carolina USA; 6 Department of Otorhinolaryngology, Union Hospital, Tongji Medical College, Huazhong University of Science and Technology, 430022 Wuhan, China; 7 School of Human Communication Disorders, Dalhousie University, B3J1Y6 Halifax, NS Canada; 8 Jiangsu Province High-Tech Key Laboratory for Bio-Medical Research, Southeast University, 211189 Nanjing, China; 9 Co-innovation Center of Neuroregeneration, Nantong University, 226001 Nantong, China; 10 Institute for Stem Cell and Regeneration, Chinese Academy of Science, Beijing, China; 11 The Second Affiliated Hospital and Yuying Children’s Hospital of Wenzhou Medical University, 112008 Wenzhou, China; 12 Department of Otolaryngology, Hangzhou First People’s Hospital, 310006 Hangzhou, Zhejiang China; 13 Department of Otolaryngology-Head and Neck Surgery, The second Xiangya Hospital, Central South University, 410011 Changsha, Hunan Province China

## Abstract

Inherited hearing loss is associated with gene mutations that result in sensory hair cell (HC) malfunction. HC structure is defined by the cytoskeleton, which is mainly composed of actin filaments and actin-binding partners. LIM motif-containing protein kinases (LIMKs) are the primary regulators of actin dynamics and consist of two members: LIMK1 and LIMK2. Actin arrangement is directly involved in the regulation of cytoskeletal structure and the maturation of synapses in the central nervous system, and LIMKs are involved in structural plasticity by controlling the activation of the actin depolymerization protein cofilin in the olfactory system and in the hippocampus. However, the expression pattern and the role of LIMKs in mouse cochlear development and synapse function also need to be further studied. We show here that the *Limk* genes are expressed in the mouse cochlea. We examined the morphology and the afferent synapse densities of HCs and measured the auditory function in *Limk1* and *Limk2* double knockout (DKO) mice. We found that the loss of *Limk1* and *Limk2* did not appear to affect the overall development of the cochlea, including the number of HCs and the structure of hair bundles. There were no significant differences in auditory thresholds between DKO mice and wild-type littermates. However, the expression of p-cofilin in the DKO mice was significantly decreased. Additionally, no significant differences were found in the number or distribution of ribbon synapses between the DKO and wild-type mice. In summary, our data suggest that the *Limk* genes play a different role in the development of the cochlea compared to their role in the central nervous system.

## Introduction

The cochlea is the primary sensory organ in the inner ear for hearing. There are two types of sensory hair cells (HCs) – inner hair cells (IHCs) and outer hair cells (OHCs) – and different supporting cells (SCs), including Deiters’ cells, pillar cells, Hensen’s cells, inner border cells, and inner phalangeal cells^1–3^ (Supplementary Fig. 1). The HCs serve as mechanosensory receptors and convert mechanical sound stimuli into electric signals^4–7^. When sound stimulation occurs, the hair bundles of HCs are deflected due to the shearing of the sensory epithelium and the tectorial membrane in the regions of the stimulus^8–10^. Deflection of the hair bundles opens mechanosensitive channels, which induces a depolarizing current that in turn induces persistent and graded receptor potentials in the HCs^11,12^. The electromotility of OHCs, which form the foundation for sound amplification in the cochlea, is largely dependent on the properties of prestin, which is a motor protein unique to mammals^13–15^. Conclusive evidence for the requirement of prestin for cochlear amplification was obtained from its genetic deletion in mice, which led to the loss of electromotility in isolated OHCs and to a 50-dB decrease in cochlear sensitivity *in vivo*^16,17^. The receptor potential of IHCs is responsible for regulating the structure of synaptic vesicles and for the release of glutamate from the ribbon synapse^18,19^. Abnormalities in the structure or function of HCs usually lead to hearing loss.

LIM motif-containing protein kinases (LIMKs) modulate actin arrangements^20–23^, and they consist of two N-terminal LIM domains, a PDZ domain, and a C-terminal kinase domain. LIMK1 and LIMK2 are the two isoforms of LIMK^24^. In the central nervous system, inhibition of LIMKs reduces actin assembly of the growth cone peripheral region of chick dorsal root ganglion neurons^25^. Transient overexpression of *Limk1* promotes axon formation, but permanent overexpression of *Limk1* damages the growth cone and leads to axon retraction^26^. Cofilin is an actin depolymerizing factor that is inactivated by phosphorylation by LIMKs, and loss of *Limk1* shows a significant decrease in the phosphorylation of cofilin in the central nervous system with clustered accumulations of actin filaments along the dendrites, which are smaller than normal^27^. Additionally, *Limk1* knockout mice show increased miniature excitatory postsynaptic currents and enhanced synaptic depression, thus highlighting the role of *Limk1* in synaptic function^28^. Previous reports show that the LIMK-mediated pathway has a profound influence on the motility of OHCs, and LIMK-mediated phosphorylation of cofilin increases both the electromotility and OHC length in guinea pigs^29,30^. *Limk1* is expressed in the mouse brain during embryogenesis^31^, and *Limk2* is expressed in the placenta, brain, and kidney^32^. However, the detailed expression pattern and the roles of *Limk1* and *Limk2* during the development of the mouse cochlea need to be further investigated. Here we report the expression pattern of LIMK1/2 in the mouse cochlea and the role of these proteins in auditory function and HC morphology in the mouse cochlea using a *Limk1* and *Limk2* double knock out (DKO) mouse model^33,34^.

## Results

### The expression of LIMKs in the mouse cochlea

**Figure 1:**
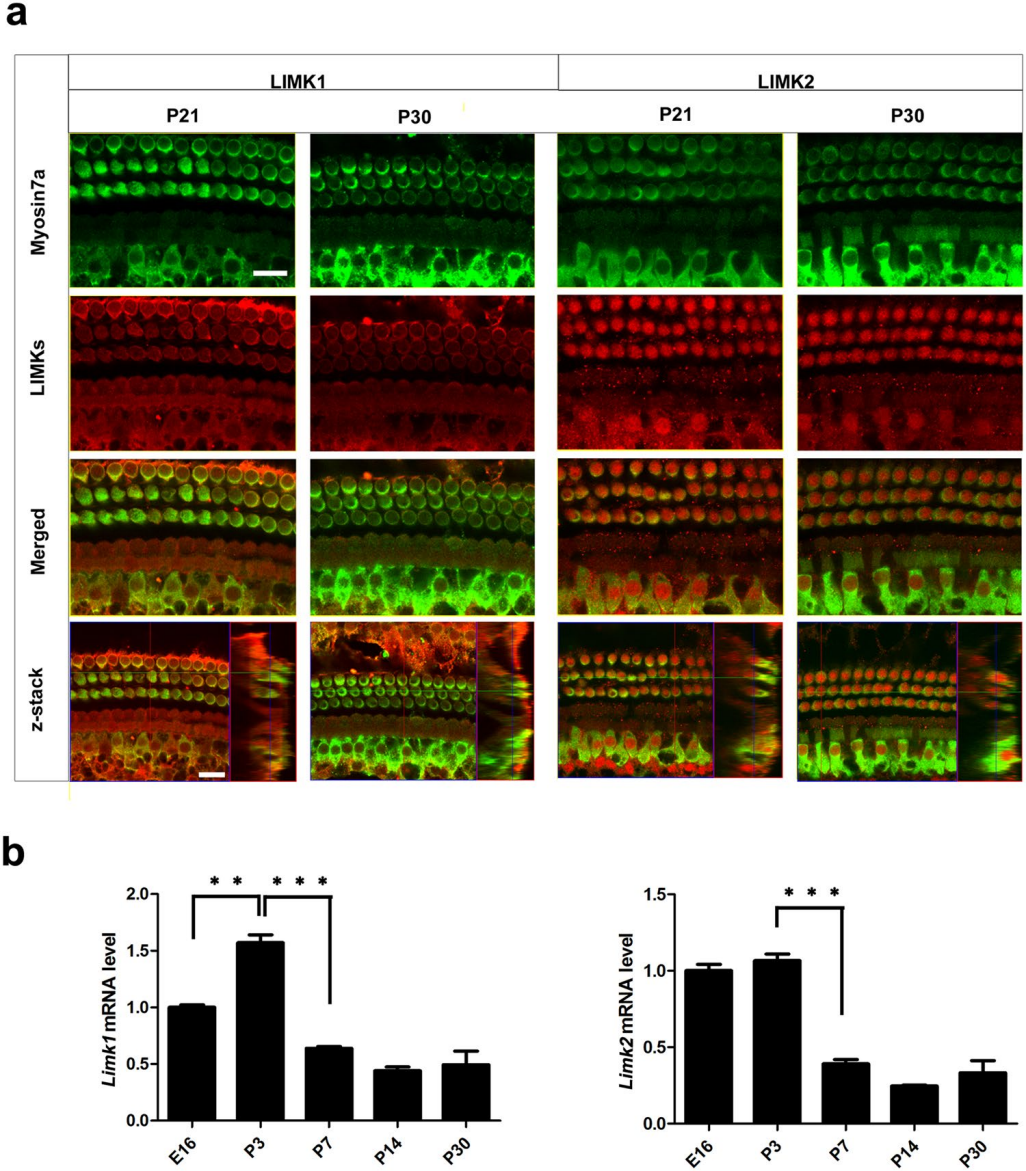
Expression of LIMKs in WT mouse cochlea. (**a**) Immunofluorescence staining showed that LIMK1 and LIMK2 were expressed in the cochlear epithelium in the P21 and P30 mice. Myosin7a was used as a marker for HCs. Images were taken from the basal turn of the sensory epithelium. There was no difference in the immunolabeling of LIMK1 and LIMK2 from the apical to basal turns. Scale bar = 10 µm. (**b**) RT-qPCR results show the changes in expression of *Limks* in the mouse cochlea from embryonic development to adult. β-actin was used as the internal control. Data are presented as mean ± SD. (*p* < 0.01, n = 5).

**Figure 2:**
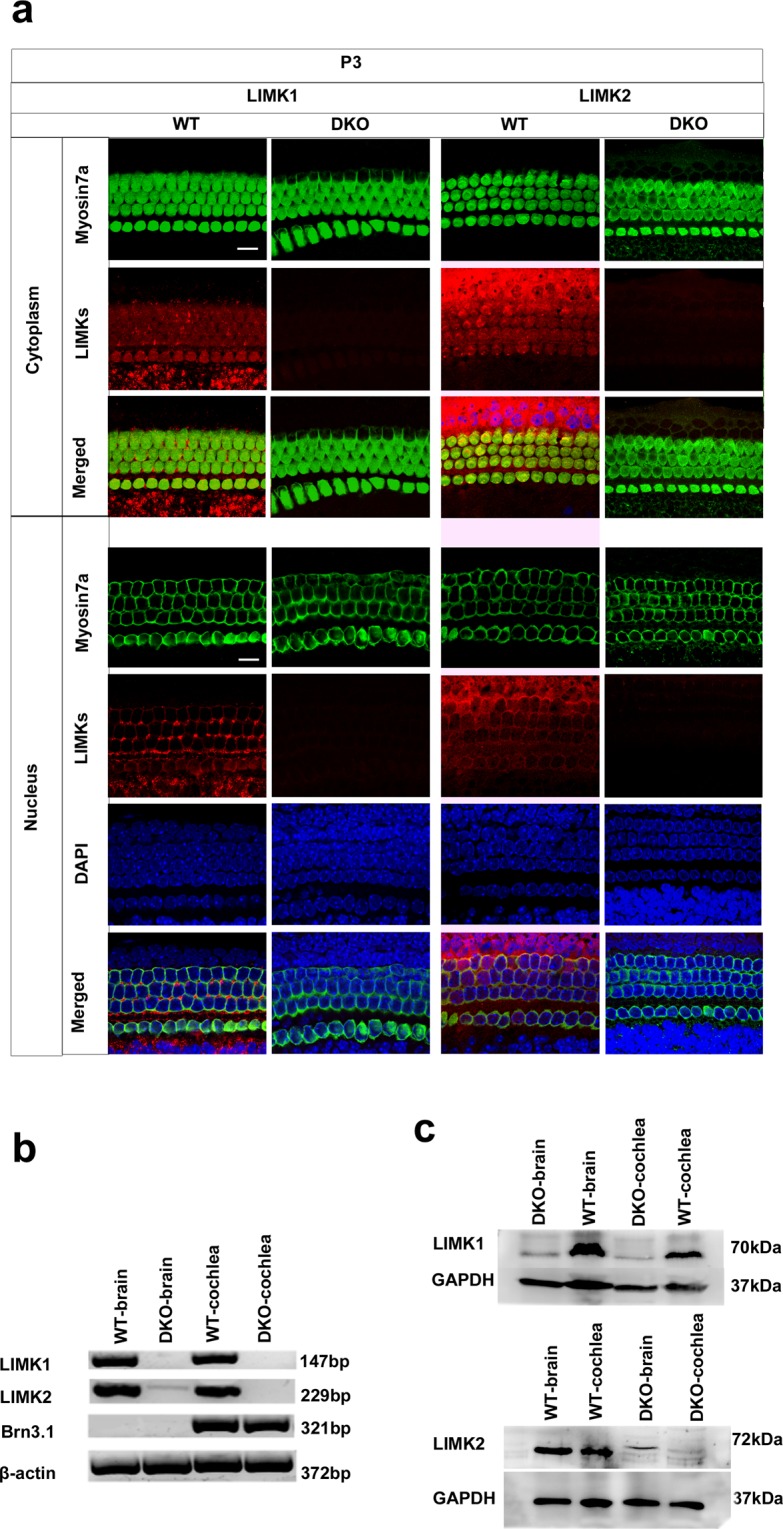
Analysis of LIMK expression in the DKO mice at the mRNA and protein level. (**a**) The cochleae of P3 DKO mice were immunolabeled with LIMK1 and LIMK2 antibodies. Scale bar = 10 µm. Images were taken from the basal turn of the sensory epithelium. There was no difference in the immunolabeling of LIMK1 and LIMK2 from the apical to basal turns. (**b**) RT-PCR was performed to analyze the DKO mice. Total cochlear RNA was extracted from P3 DKO and WT mice. Brn3.1 was used as the positive control, and β-actin was used as the internal control. (**c**) Western blot was performed to analyze DKO mice using antibodies against LIMK1 and LIMK2. Proteins from the brain and cochlea were extracted from P3 DKO and WT mice, and GAPDH was used as the internal control.

To determine the relative expression of LIMKs in the cochlea, we first immunolabeled LIMK1 and LIMK2 in the cochlear epithelia of postnatal day 21 (P21) wild-type (WT) mice with myosin7a, which is a specific HC marker with a cytoplasmic expression pattern, and sox2, which has a nuclear expression pattern and labels the SCs, including Hensen’s cells, Deiters’ cells, pillar cells, inner phalangeal cells, and inner border cells. Confocal imaging of the whole-mount organ of Corti showed that LIMK1 was mainly expressed in the cytoplasm of HCs and SCs at P21, while almost all of the LIMK2 was in the nuclei of HCs and SCs (Fig. 1a, Supplementary Fig. 2). The same expression pattern of LIMKs at P21 was seen at P30 (Fig. 1a. Supplementary Fig. 2). When looking at earlier time points, we found that the expression of LIMK2 was mainly in the cytoplasm at P3 but transferred into the nucleus during postnatal development (Fig. 2a). There was no difference in the immunolabeling of either LIMK1 or LIMK2 from the apical to basal turns, and together these results confirm that the expression pattern of LIMKs is maintained after the auditory system is fully matured. In order to confirm the changes in the expression of the *Limk* genes, q-PCR was performed in the WT cochlea. We found that *Limk1* expression was significantly increased at P3 compared with E16 and then decreased significantly during postnatal development. The expression of *Limk2* was not significantly different at P3 compared with E16, but the subsequent reduction in *Limk2* expression was similar to that of *Limk1* (*p* < 0.01, n = 5) (Fig. 1b).

### The *Limk1* and *Limk2* genes are knocked out in the DKO mouse cochlea

The DKO mice were genotyped as described previously^28,34,35^ (Table 1). The coding exon of the LIM and PDZ domains were replaced by the neomycin cassette to create the *Limk1* KO mice, and deleting exons 3–5 generated the *Limk2* KO mice. To confirm that the *Limk1* and *Limk2* genes were knocked out in the mouse cochlea, we dissected the cochleae and brains from P3 WT and DKO mice. The immunolabeling of LIMK1 and LIMK2 was seen in HCs of the WT mouse cochlea, but almost none was detected in the DKO mouse cochlea (Fig. 2a). The RT-PCR results confirmed that the *Limk1* and *Limk2* genes were knocked out in the DKO mice (Fig. 2b, Table 2). Western blots showed that specific bands with a molecular mass around 72 kDa, consistent with the expected sizes of LIMK1 and LIMK2, were detected in the cochlear and brain tissues from WT mice, while only very faint bands were seen in DKO mice (Fig. 2c). The faint signal in the DKO mice might be due to the non-specific binding of the antibody. These results confirmed that the *Limk1* and *Limk2* genes had been successfully knocked out in the DKO mouse cochleae.

**Table 1:** Genotyping primers used in the experiments.

Genes		Sequences	Product length
*Limk1*	WT-forward	CCAGACCGTGGTAACTCCAG	307 bp
	WT-reverse	CTCTTCCCCACACAGGTTG	
	Mutant -forward	GACCATGATGGAAGGAGAG	500 bp
	Mutant -reverse	GGGGGAACTTCCTGACTAGG	
*Limk2*	WT-forward	GCTCTCTCCTATATTGGAGC	358 bp
	Common	CAGAGATGTGAGACAGATAC	
			500 bp
	Mutant-forward	GTTGGGACTGATGTGCATC

**Table 2:** RT-PCR & RT-qPCR gene primers used in the experiments.

Genes	Forward	Reverse	Product length
*Limk1*	CGAACAGATCCTACCTGACTCA	GAATGCTCCGTGCCACATC	147 bp
*Limk2*	CCTACTCTGTCACGCTCATCT	GCATCCTCCACCTCCTCTAC	229 bp
*Brn3.1*	ACCCAAATTCTCCAGCCTACAC	GGCGAGATGTGCTCAAGTAAGT	321 bp
*β-actin*	ACGGCCAGGTCATCACTATTG	AGGGGCCGGACTCATCGTA	372 bp
*Ctbp2*	TCGGTAGTGGCTACGACAAC	CGCCGATACAGATTGAGAATGT	133 bp
*Psd95*	GACGGGAGTGGTCAAGGTTA	GGCGAGCATAGTGAACTTCC	120 bp
*Cofilin*	CCTCTTGGTGTGGCTGTCTCTGAT	GCGCTGCCACCTAGTTTCTCTG	467 bp
*formin*	ATGGGGAACCAGGATGGGAA	CTTGCCAAGCGCCTTTTTG	138 bp
*profilin*	AACGCCTACATCGACAGCC	CGTAATGCTAACGAAGGTCTTCC	120 bp
*tropomyosin*	GTGGCTGAGAGTAAATGTGGG	TTGGTGGAATACTTGTCCGCT	98 bp
*myosin*	GCCCTCAAGGACGAGAGGT	CTCATCTCCCGTTTCTGTGAC	197 bp
*Arp2*	TGGTGATGAGGCAAGTGAGC	ATGTGTAGTCCCACAGGTGC	107 bp
*Arp3*	CCACGCCTGAGTTCTACCAA	TCCATCTCCCTAACCCAATCAAC	136 bp
*TESK1*	CGAGCGTCGGAATCTCAACTTTA	ATCCCTGACCTGCTCGACT	71 bp
*TESK2*	GGGCTGGATTACGATGCTTTC	CTTTACAGAGCCAACCAGGG	168 bp
*Hsp25*	CAGCCTTGACCAGCCAAGAA	GAACCACTGCGACCACTCAT	162 bp
*GAPDH*	GCAAGAGAGAGGCCCTCAG	TGTGAGGGAGATGCTCAGTG	74 bp

### No alteration of general cochlear development in the DKO mice

**Figure 3:**
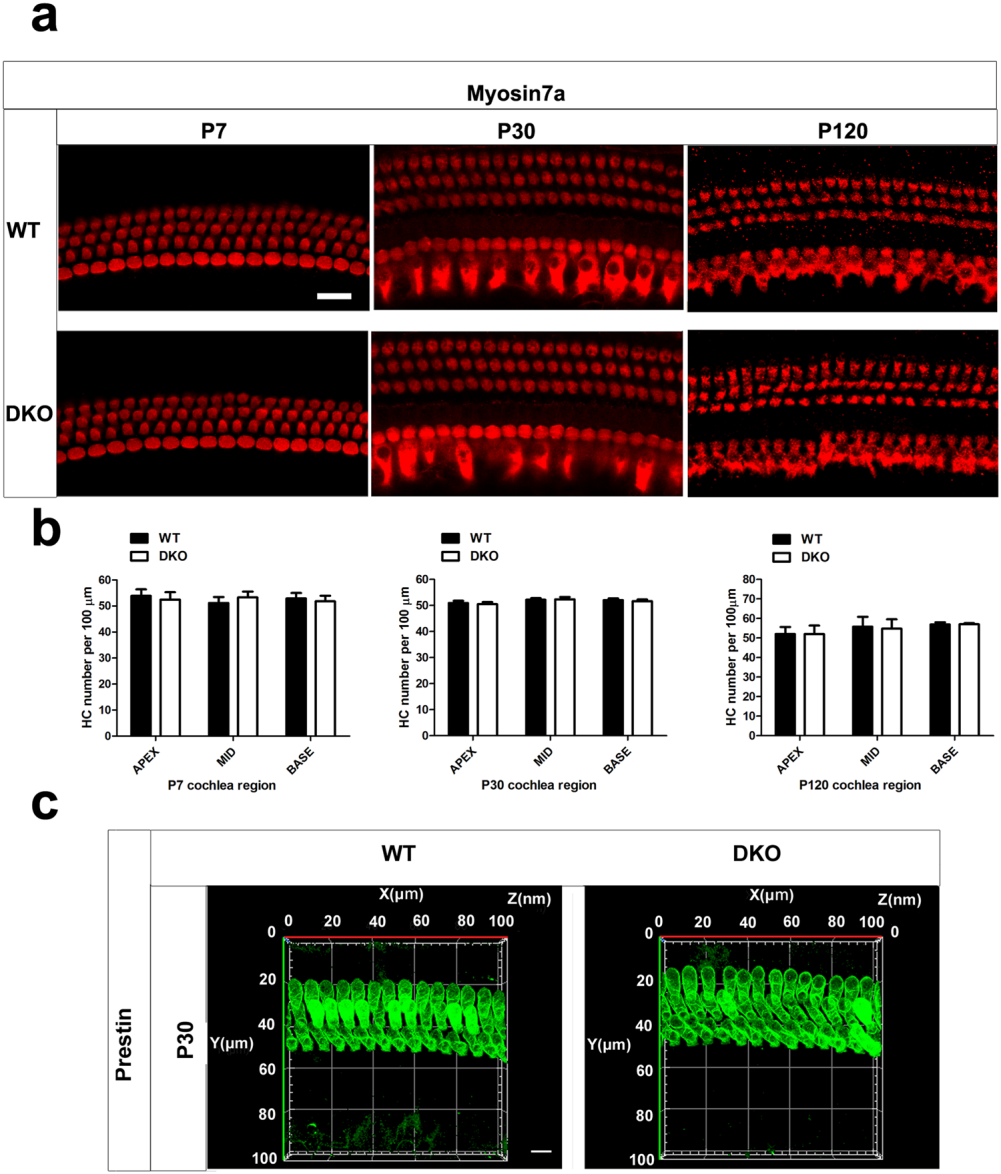
The auditory HCs are morphologically normal in the DKO mice. (**a**) Auditory HCs of P7, P30, and P120 mice were stained with antibodies against myosin7a and imaged using a confocal microscope. Scale bar = 10 µm. Images were taken from the basal turn of the cochlea. There was no difference in the staining from the apical to basal turns. (**b**) The HCs were counted and compared with age-matched WT mice (*p* > 0.05, *n* = 4). Data are presented as mean ± SD. (**c**) Auditory OHCs of P30 mice were stained with antibodies against prestin and imaged using a confocal microscope. Images were taken from the basal turn of the cochlea. Scale bar = 10 µm.

LIMK1 and LIMK2 have been suggested to play fundamental roles in cell cytoskeleton regulation, and thus we hypothesized that the DKO mice would show cochlear developmental deficits. The cochleae of P30 DKO mice were dissected, and we performed immunofluorescence reactions to examine the structure of the cochlea. Unexpectedly, the cochleae from DKO mice were morphologically indistinguishable from the cochleae of P7 and P30 WT mice, and we did not observe any significant HC loss in P7 and P30 DKO mice. Even at P120 we did not observe any significant differences in morphology or HC populations between WT and DKO mice (Fig. 3a,b, Supplementary Fig. 3). Prestin is the motor protein that is related to OHC motile activity, and in order to confirm the expression and structure of prestin, immunolabeling was performed in P30 DKO and WT mice. The expression of prestin in DKO mice was not obviously different compared to WT mice (Fig. 3c). FM1-43, as a marker of functional mechanotransduction channels in HCs, was used to investigate the HC function in DKO mice. No obvious signals were seen in either WT mice or DKO mice at P0, which indicates that the mechanotransduction channels in HCs are immature at this time. At P14, there was no difference in the signal between DKO mice and WT mice (Supplementary Fig. 5a).

### The stereocilia structures of HCs in DKO mice are intact

**Figure 4:**
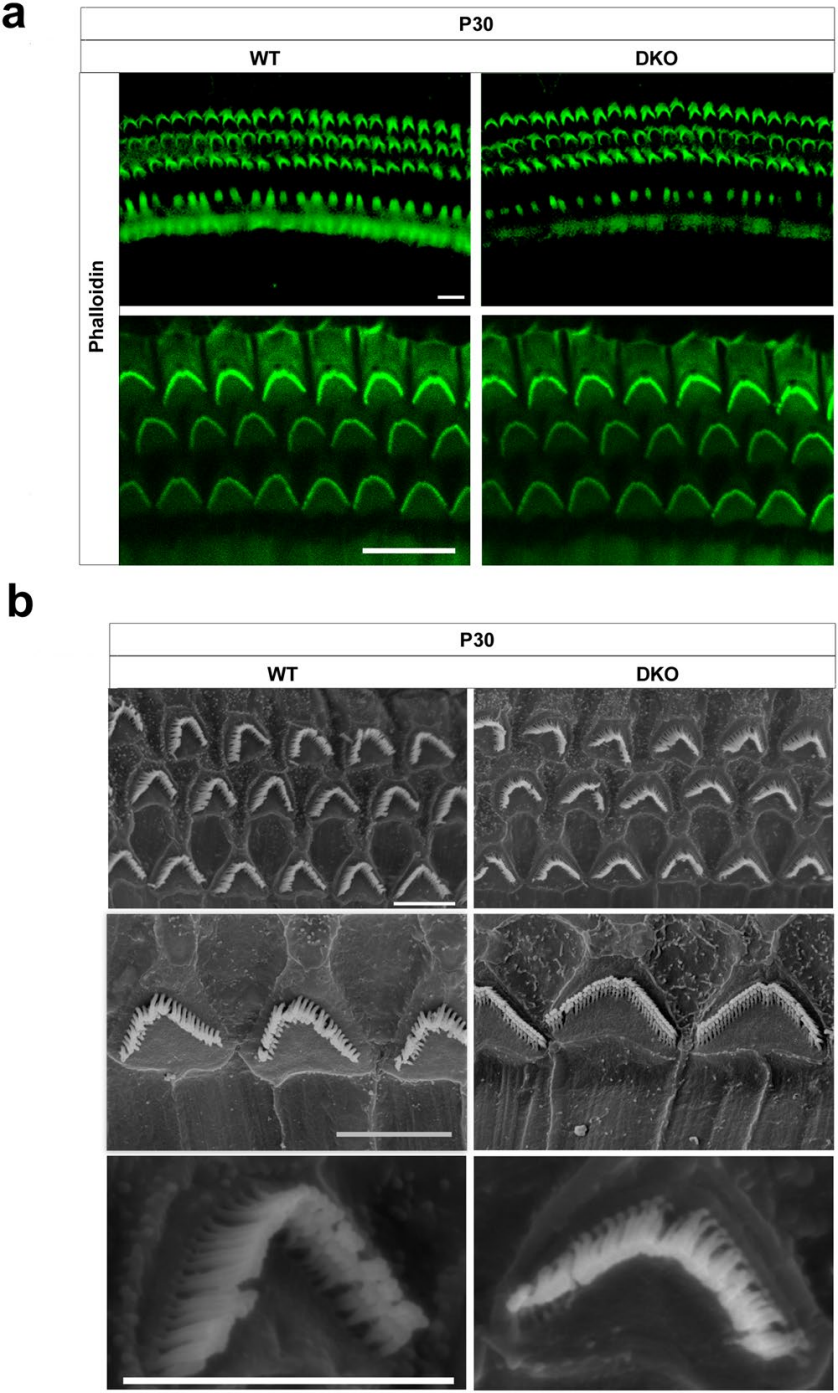
The auditory HC stereocilia are morphologically normal in DKO mice. (**a**) Auditory HC stereocilia of DKO and WT mice were stained with FITC-conjugated phalloidin and imaged using a confocal microscope. Images were taken from the basal turn of the cochlea, and there was no difference from the apical to basal turns. Scale bar = 10 µm. (**b**) Low magnification and high magnification scanning electron microscope images of OHC stereocilia bundles of DKO and WT mice. Images were taken from the middle turn of P30 mice. Scale bar = 5 µm.

Stereocilia are actin-based protrusions on sensory epithelium HCs that are deflected by sound stimuli to initiate the transformation of mechanical energy into electrical signals, and proper stereocilia structure is essential for maintaining the auditory function of HCs. Immunofluorescence staining of phalloidin showed that the stereocilia polarity of P30 cochlear HCs in DKO mice was normal (Fig. 4a). We then performed scanning electron microscopy to examine the structure of the cochlear HC stereocilia at higher resolution, but still no differences were seen between DKO and WT mice along the tonotopic regions (Fig. 4b). These results suggest that the knockout of LIMKs does not affect the morphology of cochlear HC stereocilia in mice.

### Knockout of *Limk1* and *Limk2* does not change IHC ribbon synapses

**Figure 5:**
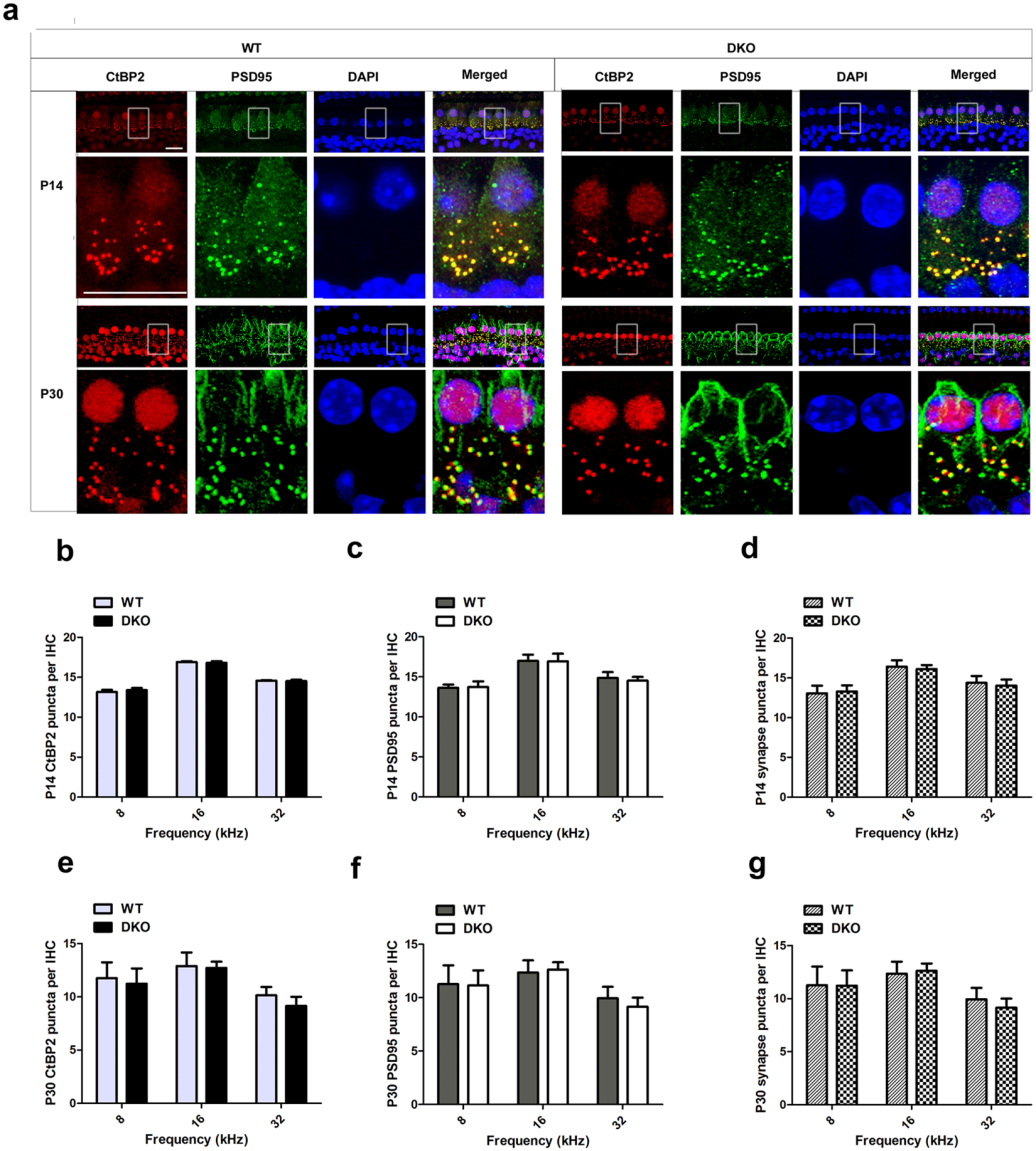
The ribbon synapses were normal in DKO mice. (**a**) Ribbon synapses of P14 and P30 DKO and WT mice were stained with the ribbon synapse-specific markers CtBP2 and PSD95 and imaged under a confocal microscope. Images were taken from the basal turn of the cochlea, and there was no difference from the apical to basal turns (**b**,**c**,**e**,**f**). The total numbers of synapses from the 8-kHz to 32-kHz region were counted and compared between WT and DKO mice (**d**,**g**). The numbers of functional synapses in DKO mice were compared with WT mice. No significant differences were seen for any measurements (*p* > 0.05, n = 4). Scale bar = 10 µm. Data are presented as mean ± SD.

Because p-cofilin regulates the length of the postsynaptic density of asymmetric synapses in the hippocampus of the mouse^36^, we assessed whether there was any potential change in the molecular composition and/or amount of cochlear ribbon synapses in the HCs of the P14 and P30 DKO mice by immunolabeling the pre-synaptic ribbons and post-synaptic ribbon terminals with antibodies against CtBP2 and PSD95, respectively (Fig. 5a, Supplementary Fig. 4). As specialized components of the pre-synaptic ribbons, the ribbon synapses contain three members of transcriptional repressors: CtBP1, CtBP2, and an alternative splice form called RIBEYE. The signals of CtBP2 were seen in the lateral aspects of the IHCs^37^. Consistent with a previous study, the PSD95 signal was concentrated at the terminus and the plasma membrane of IHCs^38^, and this staining did not interfere with the detection of the target signal. The red dot-like staining of CtBP2 at the base of the IHCs overlapped with the PSD95 signal. The synapse densities of the IHCs were comparable between DKO mice and WT controls in all three turns of the cochlea, and no significant differences were seen in the amount of CtBP2 and PSD95-positive puncta in DKO mice compared to WT mice (Fig. 5b,c,e,f). Because nerve fibers will not trigger an action potential when pre-synapses are disconnected from post-synapses, we analyzed the co-localization of CtBP2 and PSD95. We found that almost all of the CtBP2 puncta were overlapping with PSD95 in both WT and DKO mice (Fig. 5d,g). Together, our results indicate that knockout of LIMKs did not change cochlear IHC synapses during postnatal development.

### Phosphorylated cofilin is significantly decreased in P30 DKO mice

**Figure 6:**
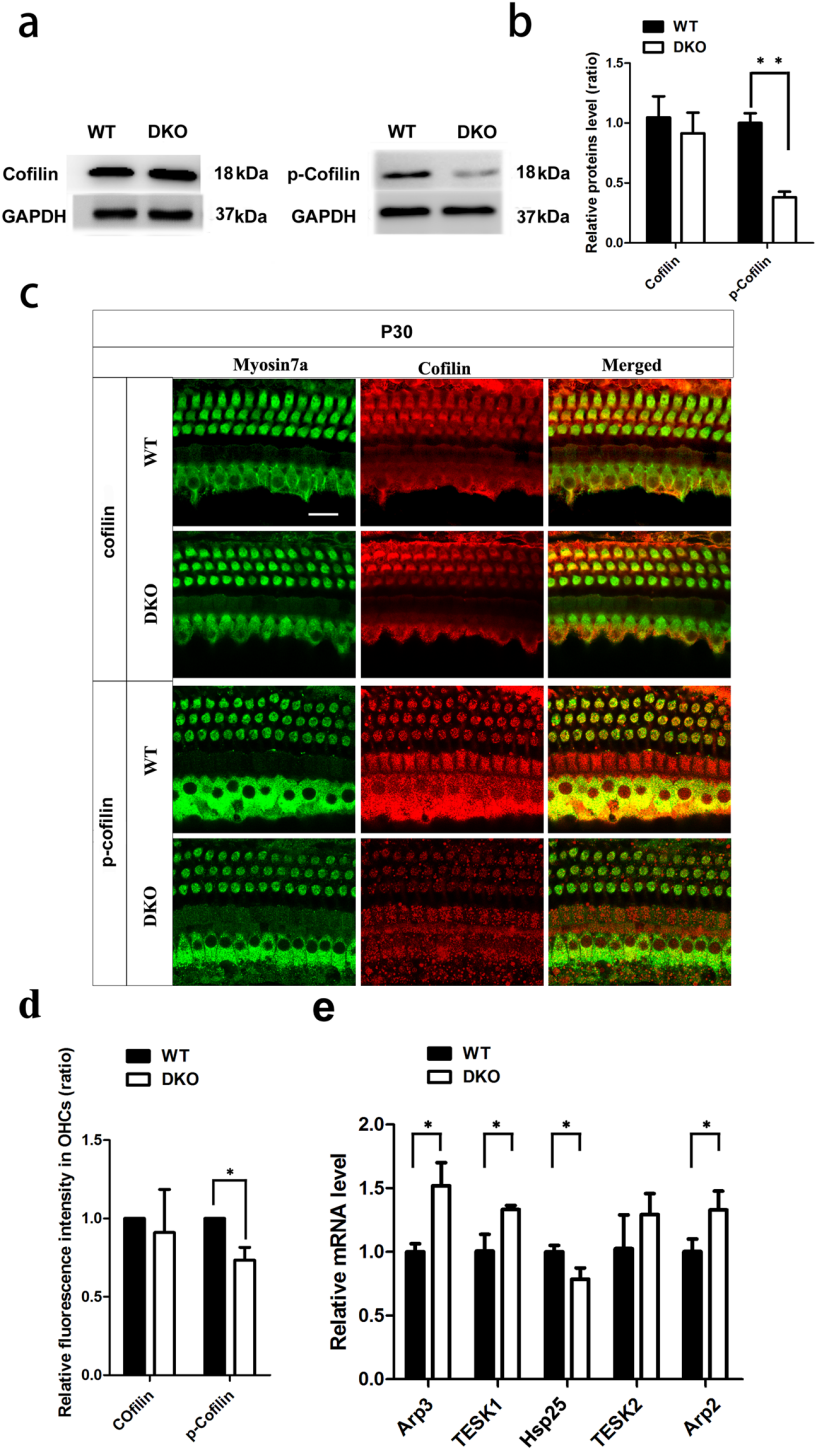
The effect of loss of LIMKs on cofilin. (**a**) Western blot was performed to detect the expression level of cofilin and p-cofilin in both P30 WT and DKO mice, n = 3. (**b**) Quantification of the Western blot (*p* < 0.01). (**c**) Auditory HCs of P30 DKO and WT mice were stained with cofilin and p-cofilin antibodies. Images were taken from the basal turn of the cochlea, and there was no difference from the apical to basal turns. Scale bar = 10 µm. (**d**) Quantification of cofilin and p-cofilin immunolabeling in the basal turns of OHCs (*p* < 0.05) n = 3. (**e**) RT-qPCR was performed with P30 WT and DKO mice cochleae (*p* < 0.05, n = 5). Data are presented as mean ± SD.

Because cofilin, which is regulated by LIMKs, has a strong influence in the formation of synapses in mature neurons in the hippocampus^39,40^, we analyzed the expression of cofilin and p-cofilin by Western blot and immunofluorescence in P30 DKO and WT mice. The Western blot data suggested that the levels of cofilin were similar between the DKO and WT mice (Fig. 6a,b), but the expression of p-cofilin was significantly decreased in the DKO mice (*p* < 0.05, *n* = 4) (Fig. 6a,b). The immunostaining data also showed that the immunofluorescence intensity of cofilin in HCs was similar between the DKO and WT mice (Fig. 6c,d), but the immunofluorescence intensity of p-cofilin was significantly decreased in the DKO mice (*p* < 0.05 *n* = 3) (Fig. 6c,d). In order to determine if knockout of LIMKs altered other synapse-related or actin-related genes, we measured the mRNA levels of synapse-related genes and actin-related genes using RT-qPCR in the cochleae of P30 WT and DKO mice. *Cofilin*, *formin*, *profilin*, *myosin*, *tropomyosin*, *CtBP2*, and *PSD95* showed no significant differences compared with WT mice (Supplementary Fig. 5b), while the expression of *Arp2, Arp3*, and *TESK1* were significantly increased and *Hsp25* was significantly decreased in DKO mice compared with WT controls (*p* < 0.05 *n* = 5) (Fig. 6e).

### DKO mice show no changes in auditory function

**Figure 7:**
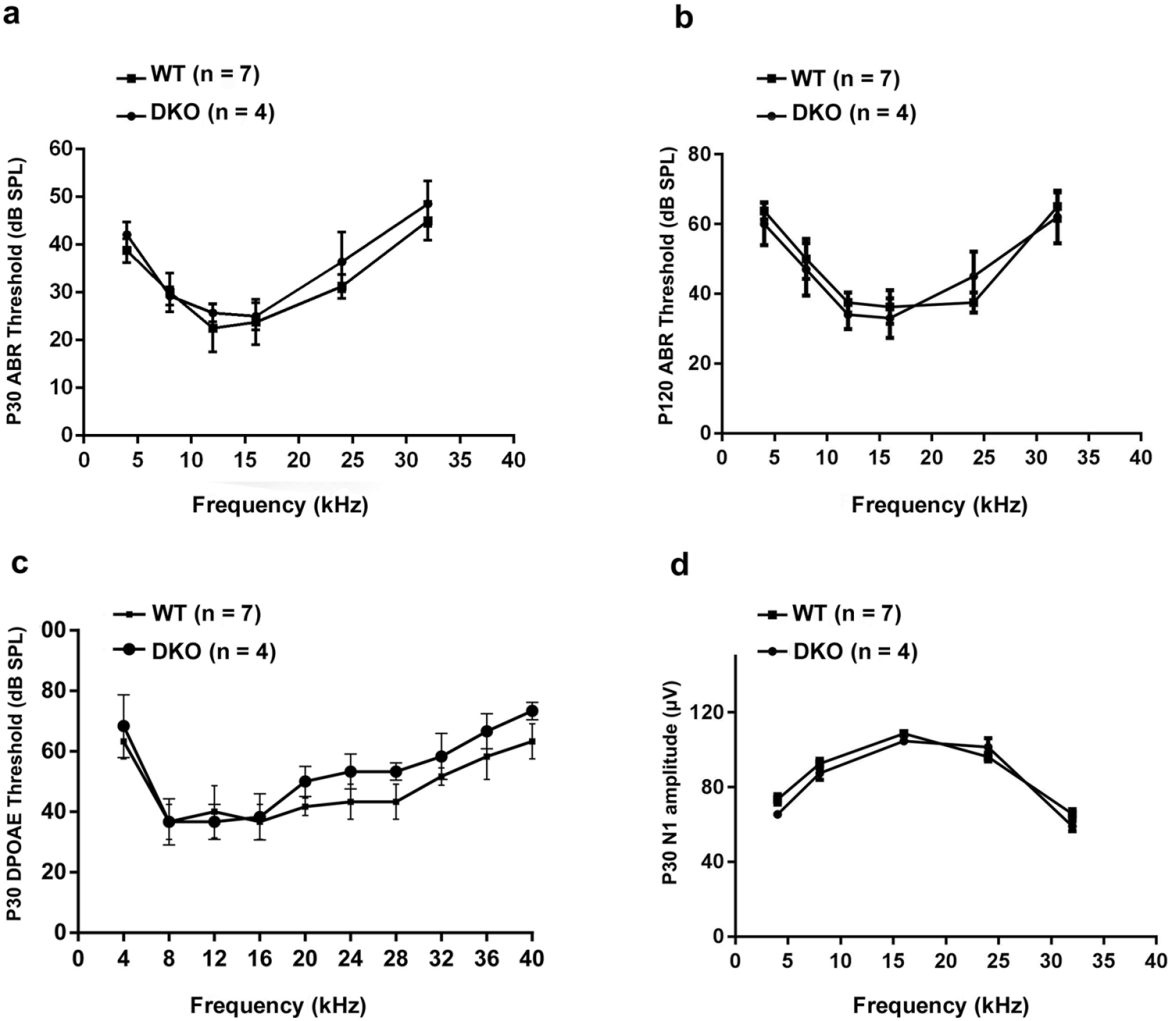
Auditory measurements show normal auditory function in DKO mice. (**a**) ABR thresholds of P30 DKO and WT mice were measured at 4, 8, 12, 16, 24, and 32 kHz. (**b**) ABR thresholds of P120 DKO and WT mice were measured at 4, 8, 12, 16, 24, and 32 kHz. (**c**) DPOAE thresholds were measured in response to tone bursts of 4, 8, 16, 20, 24, 28, 32, 36, and 40 kHz in P30 DKO and WT mice. (**d**) CAP amplitudes of P30 DKO and WT mice were measured in response to tone bursts of 4, 8, 16, and 32 kHz at 90 dB SPL. No significant differences were observed between DKO and WT mice (*p* > 0.05). Data are presented as mean ± SD.

Although DKO mice have been reported to be generally healthy^35^, their auditory function has remained unknown. We therefore first measured their auditory brainstem response (ABR). Auditory thresholds showed no difference between the DKO mice and WT controls at P30 (Fig. 7a) or P120 (Fig. 7b). The ABR reflects the overall activity of the neural output of the cochlea, while the distortion-product otoacoustic emissions (DPOAEs) depend only on the normal function of the OHCs^41^. We measured the DPOAE of P30 DKO mice, and no obvious differences were found in DPOAE thresholds compared with WT mice (Fig. 7c). Finally, we measured the compound action potential (CAP) of different frequencies at 90 dB in response to tone bursts, in which the N1 amplitude provides a physiological representation of the mouse audiogram^42^. We found no difference in cochlear sensitivity at frequencies between 4 kHz and 32 kHz between DKO and WT mice (Fig. 7d). Together these results show that the DKO mice have full auditory function.

## Discussion

LIMKs modulate the architecture of the cytoskeleton through the phosphorylation of the cofilin family of proteins^43^, which consists of non-muscle type cofilin-1, muscle type cofilin-2, and actin depolymerization factor (ADF). This inactivation of cofilin is modulated by a number of extracellular signals, for example, LIMKs, TES kinases, and Nck-interacting kinase-related kinase^44^. Cofilin is phosphorylated at Ser-3 by several kinases^45^, and the major kinases of cofilin are LIMKs, testicular protein kinase 1/2^46,47^, and the Nck-interacting kinase-related kinase^48^ in mammals. P-cofilin is dephosphorylated in mammals by the three members of the slingshot protein phosphatase family, SSH1, SSH2, and SSH3^49–51^.

LIMK1 is known to play an indispensable role in dendritic spine function and synapse function, and previous studies suggested that *Limk1* KO mice have severely impaired long-term hippocampal potentiation and severely impaired dendritic spine morphology^28,52,53^. LIMKs might contribute to synaptic regulation through the modulation of plasticity and cellular processes, and actin is actively involved in the regulation of synaptic plasticity and signaling through presynaptic neurotransmitter release. Previous studies also showed that 15–20% of the p-cofilin was still present in DKO mice, which indicates that other compensatory signaling pathways are capable of regulating cofilin phosphorylation in the central nervous system^35^. Because LIMKs have an indispensable role in the development of the brain, we hypothesized that LIMKs would have a significant effect on the development and function of the mouse cochlea. A previous study used a patch clamp to isolate guinea pig OHCs and performed immunostaining of LIMKs in guinea pig OHCs *in vitro*, and they found that both LIMKs were abundant in both the cytoplasm and nuclei of OHCs^30^. In this study, we found that LIMKs were expressed in the WT mouse cochlea by whole-mount immunostaining. However, most of the antibody signal was seen in the cytoplasm of HCs and SCs, while the signal for LIMK2 antibody was mainly seen in the nuclei of HCs and SCs in the adult mice (Fig. 1, Supplementary Fig. 2). The difference in expression pattern might due to the different animal species or due to the different immunostaining methods.

The different subcellular locations of LIMK1 and LIMK2 might indicate distinct roles of LIMKs in HCs. Although knockout of both LIMK1 and LIMK2 proteins in the DKO mice decreased the levels of p-cofilin in the mouse cochlea, the auditory function and structural arrangements of hair bundles and ribbon synapses showed no alterations compared to age-matched WT mice up to P120. However, it is possible that older DKO mice might have more severe hearing loss compared to WT controls. As for aging DKO mice, whether the loss of LIMK1 and LIMK2 will cause hearing loss is still unknown and needs to be further studied. Moreover, the effect of LIMKs in the development of dendritic spines on auditory nerve dendrites was not investigated in the current study and should be investigated in the future.

The actin dynamics are not only indispensable for maintaining the cytoskeleton and for extending cellular protrusions, but are also indispensable for internal cell rearrangements. The mechanical forces that drive intracellular processes are provided by actin filament formation at intracellular membranes. The dynamics of the actin cytoskeleton require the formation of rod-like actin polymers, known as filaments, from actin monomers. The process by which G-actin is removed from the trailing end of an actin filament and is added onto the opposing fast-growing end of F-actin is referred as actin treadmilling^54^, and the cofilin/ADF proteins are critical intermediates that rely on the upstream proteins to modulate actin filament assembly.

Prestin is a tetrameric protein expressed in the lateral wall plasma membrane of OHCs and is necessary for the electromotility of OHCs. Prestin has been under intense investigation due to its crucial role in cochlear amplification, which enhances sound-induced vibration in the cochlea to activate the mechanical processes behind hearing sensitivity. Prestin is located in the lateral wall membrane of OHCs, along the region of a well-organized actin-based cortical cytoskeleton^15^. Prestin KO mice lack an organized cytoskeleton, suggesting that prestin is essential for maintaining such structures^55^. However, in our study we did not find an auditory phenotype in the DKO mice, and this indicates that the motility of OHCs in DKO mice was not significantly different from WT. Also, there were no obvious differences in the amount of functional mechanotransduction channels between DKO mice compared to WT. The depolymerization of actin plays an important role in the modulation of the motility of OHCs through the ROCK-LIMK1 signaling pathway. Furthermore, the active ROCK-PKCα signaling cascade with lysophosphatidic acid leads to phosphorylation of Ser-726, which is well known as an actin-spectrin dissociation promoter that regulates the length of OHCs and enhances the OHCs’ electromotile amplitude. Thus, we also speculate that the ROCK-PKCα pathway and the ROCK-LIMK1 pathway in OHCs engage in active cross talk and work in parallel with each other^29,30^. ROCK-PKCα might also be a compensatory signaling pathway for OHC motility in the DKO mice.

Cofilin contributes to the dynamics of actin filaments. Cofilin promotes depolymerization at low concentrations but promotes nucleation at high concentrations^56^. Previous studies in the central nervous system showed that diminished expression of p-cofilin induces synaptic loss in the hippocampus^36,57^. However, although the expression of p-cofilin protein is significantly decreased in the DKO mice, this decrease does not change cochlear development or auditory function in the mice, which is different from the previous reports of the effect of cofilin in the central nervous system^36,57^. Several possible reasons might explain the difference between the effect of cofilin in the brain and auditory system. First, there are several important regulators of the cytoskeleton, including the actin nucleator formin, the actin-sequestering protein profilin, the actin-related protein 2/3 complex^58^, ADF/cofilin^46,58^, the actin motor protein myosin^59^, and the actin-stabilizing protein tropomyosin^60^. Thus, cofilin might not be the key factor regulating the actin cytoskeleton in the inner ear. This hypothesis is supported by our qPCR data. We did not find any statistical difference in synapse-related genes (*CtBP2* and *PSD95*) or some actin-related genes (*cofilin*, *formin*, *profilin*, *myosin*, and *tropomyosin*) between DKO and WT mice, indicating that the homeostasis between actin polymerization and depolymerization is unchanged in the DKO mice. Second, the p38/MK2/Hsp25 and Cdc42/PAK/LIMK1 signaling pathways work in parallel to modulate specific actin-related proteins in mesenchymal cells^61,62^. RhoA/ROCK/PKCα might be a compensatory signaling pathway that regulates the motility of OHCs. Thus, these alternative signaling pathways might modulate the cytoskeleton in the inner ear and compensate for the lack of the LIMK proteins. The Arp2/3 complex, which can be phosphorylated by p21-activated kinase 1 (PAK1) to promote actin polymerization, was significantly increased in DKO mice^63^. TESK1 is another signaling molecule working in parallel to LIMKs and can also phosphorylate cofilin to inhibit the depolymerization of actin^64^. The significant elevation of *TESK1* expression seen here indicates that this compensatory signaling plays a role in actin dynamics. The phosphorylation of Hsp25 has been associated with modification of the actin cytoskeleton^61^, and the reduction of Hsp25 might also be a compensatory pathway for the regulation of actin dynamics (Fig. 6e). Third, cofilin and ADF play redundant roles during the development of the kidney, and conditional knockout of *cofilin-1* in the ureteric buds of mice and the Wolffian ducts did not cause an obvious effect during the development of renal morphology, which is in agreement with our findings^44^. Our study shows that the ratios of cofilin and ADF differed in different cell types and at various developmental periods. Thus, the decrease in p-cofilin levels seen in our DKO mice might be compensated for by other mechanisms that depend on the concentration of cofilin. This might also explain why no significant defects in cochlear development or auditory function were seen in DKO mice in spite of the fact that the p-cofilin level in DKO mice decreased significantly. Rac and Cdc42 are upstream regulators of LIMKs, and the lack of Rac or Cdc42 causes disorganization of hair bundles. These processes can also be controlled by Lis1, which is a potential upstream regulator of both Cdc42 and Rac1^65,66^; however, as one of their downstream effectors, LIMKs should also have negative effects on actin dynamics. There are around 40 known eukaryotic LIM proteins in different species based on the presence of LIM domains. The LIM superclass of genes is divided into 14 classes – LMO7, ENIGMA, LIMK, LASP, LHX, MICAL, PXN, EPLIN, ABLIM, CRP, LMO, PINCH, TES, and ZYX – any of which might compensate for the knockout of *Limk1* and *Limk2* in DKO mice.

In summary, our results demonstrate the expression patterns of LIMK1 and LIMK2 in the mouse cochlea. Knockout of *Limk1* and *Limk2* in DKO mice significantly decreased the level of p-cofilin in the cochlear tissue, but because of the compensatory role of *Arp2/3*, *TESK1*, and *Hsp25*, the loss of the LIMK proteins did not significantly change the number and function of ribbon synapses or the overall cochlear structure or auditory function.

## Material and Methods

### Animals and genotyping

The *Limk1* and *Limk2* DKO mice were the kind gift of Dr. Zikai Zhou of Southeast University, China. The genotyping of the transgenic mice was performed as described previously^35^. Primers used for genotyping are shown in Table 1. All mouse experiments were conducted following protocols approved by the Animal Care and Use Committee of Southeast University and were agreement with the National Institutes of Health Guide for the Care and Use of Laboratory Animals. All efforts were performed to minimize the number of animals used in these experiments and to prevent their suffering.

### Immunohistochemistry

The cochleae were placed in the medium containing 3 mM FM1-43 (Thermo Fisher, F35355) for 90 s and washed three times in PBS (pH 7.2). The cochleae then were dissected and fixed with 4% polyoxymethylene for 1 h and permeabilized with 0.5% Triton X-100 for 1 hour. The sensory epithelia were then incubated with the following primary antibodies overnight at 4 °C: anti-LIMK1 (Santa Cruz, sc-8387); anti-LIMK2 (Santa Cruz, sc-5577); anti-MYO7A (Proteus Bioscience, 25-6790); anti-SOX2 (Santa Cruz, sc-17320, sc-365823); anti-CtBP2 (C-terminal-binding protein 2), anti-IgG1 (BD Biosciences, 612044), anti-PSD95 IgG2a (Millipore, MAB1596), anti-cofilin (Abcam, ab42824), anti p-cofilin (Santa Cruz, sc-12912), and anti-prestin (Santa Cruz, sc-22692). Phalloidin (Invitrogen, A34055) was used to stain the actin cytoskeleton, and DAPI was used to label the nuclei. The tissues were washed three times with PBST (0.1 M phosphate buffer, pH 7.2, with 0.1% Triton X-100) and incubated for 1 h (37 °C) with DAPI (Sigma-Aldrich, D9542) and suitable secondary antibody (Abcam: ab150075, ab150105, ab150074, ab150073, ab150107; Invitrogen: A21131, A21124). Finally, the sensory epithelia were mounted on glass slides with Fluoromount-G mounting medium. Images were taken using a Zeiss LSM700 confocal microscope.

### Auditory measurement

A TDT System III workstation running SigGen32 software (Tucker-Davis Technologies, USA) was used to test mice for ABR and CAP recordings as previously described^3,14,67^. Mice were anesthetized using 10 mg/kg xylazine and 80 mg/kg ketamine, and the body temperature was kept at 38 °C with a thermostatic heating pad. Three subdermal electrodes were used to measure the ABRs. The reference and grounding electrodes were inserted subdermally behind the ears, and the recording electrode was inserted at the vertex of the skull. In order to confirm that the equipment worked well, the ABR threshold of mice after 120 dB noise exposure for 2 hours was used as the positive control. In order to record CAPs, the reference and grounding electrodes were inserted behind the ears and a silver electrode was placed on the round window membrane. The TDT hardware and software (BioSig and SigGen) were used to generate stimuli and record the signal. The acoustic stimuli parameters were as follows: 10 ms tone bursts for ABR and CAP with a 0.5 ms rise/fall time and cos^2^ gating. The stimuli were played using a broadband speaker (MF1; TDT) that was kept 10 cm in front of the head of the mouse. ABR thresholds were tested at 4, 8, 12, 16, 24, and 32 kHz. For each frequency, the test was measured in a downward sequence beginning with 90 dB SPL and decreasing in 5 dB steps until the ABR response disappeared. The threshold was judged as the lowest level at which a stable wave III response could be seen and repeated in the ABR test. We usually focused on the peak-to-peak amplitude, which was performed at 90 dB SPL to generate I/O functions at 4, 8, 16, and 32 kHz in the CAP test. For DPOAE, the primary tones f1 and f2 were generated and shaped using the BioSig software and TDT hardware. DPOAEs were recorded in the form of level/frequency functions at 4, 8, 12, 16, 20, 24, 28, 32, 36, and 40 kHz, and the surrounding noise floor and 2f1-f2 DPOAE amplitudes were subtracted. The DPOAE threshold was calculated offline as a way of identifying and interpolating the data when the signal was at least 5 dB SPL and greater than two standard deviations above the noise floor. If no DPOAE response was recorded even at our equipment’s limitation of 90 dB SPL, we defined the threshold as 90 dB.

### Scanning electron microscopy (SEM)

The cochlear specimens from P30 mice were fixed in 2.5% glutaraldehyde (Sigma-Aldrich, G5882) diluted in 0.1 M phosphate buffer (pH 7.2). A hole was poked in the apical region of the cochlea, and the fixative was flushed through the round window and oval window. The cochleae were kept for 24 hours at 4 °C, washed three times in PBS, and then decalcified for 3 hours in 0.5 M EDTA. The tissues were post-fixed for 2 hours at 4 °C in 1% osmium tetroxide and dehydrated in ethanol and critically point dried with liquid CO_2_ (CPD300, Leica). After applying an electrically conductive coating, the cochlear tissues were imaged on a Quanta 250 FEG scanning electron microscope.

### RT-qPCR

Total cochlear RNA was extracted with ExTrizol Reagent (Protein Biotechnology, PR910), and reverse transcription of mRNA to cDNA was performed with a cDNA synthesis kit according to the manufacturer’s instructions (Thermo Fisher Scientific, K1622). The qPCR was performed using an Applied Biosystems CFX96 qPCR system (Bio-Rad, Hercules, CA, USA) and the SYBR Green (Rox) qPCR Master Mix (Roche Life Science, 04913850001). Specific primers were designed for target DNA or mRNA. The qPCR protocols were set as follows: 15-second denaturation at 95 °C followed by 40 cycles of 15-second denaturing at 95 °C, 60 seconds annealing at 60 °C, and 20-second extension at 72 °C. The expression of mRNA was normalized using the values of *β-actin* and *Gapdh*. The results were analyzed using the comparative cycle threshold (ΔΔCt) method.

### Western blot

Cochleae from around 10 mice were dissected in cold PBS, and the cells were lysed with RIPA Lysis Buffer (Protein Biotechnology, PP109) and Cocktail-1 (Roche, 4906845001) and Cocktail-2 (Roche, 04693132001) at 4 °C. The protein concentration was measured with a BCA protein quantification kit (Protein Biotechnology, PP20). GAPDH was used as the reference protein. Donkey anti-goat immunoglobulin G (Abcam, ab6789, ab6721), peroxidase-conjugated goat anti-rabbit, and goat anti-mouse were used as the secondary antibodies. Polyvinylidene fluoride membranes were used to bind proteins, and the target proteins were detected using the SuperSignal West Dura chemiluminescent substrate kit (Thermo Scientific, 34075). Semi-quantification of the Western blot band was performed using Image J software. The background values were subtracted and the resulting signal was normalized to the GAPDH intensity and the relative optical density ratio was calculated. Each experiment was repeated at least three times.

### Cell and synapse quantification

To quantify the immunopositive cells, the entire sensory epithelia were divided into three turns. For synapse quantification, immunolabeled puncta of CtBP2 and PSD95 in IHCs were quantified from the images. Each image was acquired at 63× magnification under identical *z*-stack conditions with 1-µm intervals. The binary file was converted to a 3D *z-y* projection to measure ribbon dispersion. The synapses were manually counted one by one from the 8 kHz to 32 kHz (with each image containing ~12 IHCs) region of sensory epithelia. The number of functional synapses were manually counted by visualizing the presence of the overlapping CtBP2 and PSD95 signals.

### Statistical analysis

All of the data in this study are shown as the mean ± SD, and all experiments were repeated at least three times. Statistical analyses were performed using Microsoft Excel and GraphPad Prism 6 software. For the cell counting experiments, *n* represents the number of mice. Two-tailed, unpaired Student’s *t*-tests were performed to determine the statistical differences between two groups, and one-way ANOVA followed by a Dunnett’s multiple comparisons test was conducted when comparing more than two groups. A value of *p* < 0.05 was considered to be statistically significant.

## Supplementary information

Supplementary Information
