# Supplementary material for: Deletion of Limk1 and Limk2 in mice does not alter cochlear development or auditory function
Source: Sci Rep. 2019 Mar 4;9:3357. doi: 10.1038/s41598-019-39769-z (PMC6399249; doi:10.1038/s41598-019-39769-z)
Supplement: Supplementary file 1 — Supplementary Information [file 41598_2019_39769_MOESM1_ESM.pdf]

Deletion of *Limk1* and *Limk2* in mice does not alter cochlear development or auditory function

Qiaojun Fang[a,b,e#], Yuhua Zhang[b#], Peng Da[c#], Buwei Shao[b], Haolai Pan[d,k], Zuhong He[b,f], Cheng Cheng[b], Dan Li[b], Jiaqi Guo[b], Xiaohan Wu[b], Ming Guan[l], Menghui Liao[b], Yuan Zhang[b], Suhua Sha[e], Zikai Zhou[b], Jian Wang[d,g], Tian Wang[m], Kaiming Su[d]*, Renjie Chai[b,h,i,j]*, Fangyi Chen[a]*

[a] Department of Biomedical Engineering, Southern University of Science and Technology, Shenzhen 518000, China.
[b] Key Laboratory for Developmental Genes and Human Disease, Ministry of Education, Institute of Life Sciences, Southeast University, Nanjing 210096, China.
[c] Department of Otolaryngology-Head and Neck Surgery, Affiliated Hospital of Nantong University, Nantong 226001, China.
[d] Department of Otolaryngology, Affiliated Sixth People's Hospital, Shanghai Jiao Tong University, 600 Yishan Road, Shanghai 200233, China.
[e] Department of Pathology and Laboratory Medicine, Medical University of South Carolina, Charleston, South Carolina 29425, USA.
[f] Department of Otorhinolaryngology, Union Hospital, Tongji Medical College, Huazhong University of Science and Technology, Wuhan 430022, China.
[g] School of Human Communication Disorders, Dalhousie University, Halifax, NS, B3J1Y6, Canada.
[h] Jiangsu Province High-Tech Key Laboratory for Bio-Medical Research, Southeast University, Nanjing 211189, China.
[i] Co-innovation Center of Neuroregeneration, Nantong University, Nantong 226001, China.
[j] Institute for Stem Cell and Regeneration, Chinese Academy of Science, Beijing, China
[k] The Second Affiliated Hospital and Yuying Children's Hospital of Wenzhou Medical University, Wenzhou 112008, China.
[l] Department of Otolaryngology, Hangzhou First People's Hospital, Hangzhou, Zhejiang 310006, China
[m] Department of Otolaryngology-Head and Neck Surgery, The second Xiangya Hospital, Central South University, Changsha, Hunan Province, 410011, China.

# These authors contributed equally

***Corresponding authors:**
Renjie Chai, Ph.D., Co-Innovation Center of Neuroregeneration, Key Laboratory for Developmental Genes and Human Disease, Ministry of Education, Institute of Life Sciences, Southeast University, Nanjing 210096, China, Tel/Fax: 86-25-83790971, Email: renjiec@seu.edu.cn
Fangyi Chen, Ph.D., Department of Biomedical Engineering, Southern University of Science and Technology, 1088 Xueyuan Avenue, Nanshan Distr., Shenzhen, Guangdong 518000, China. Email：chenfy@sustc.edu.cn
Kaiming Su, M.D., Ph.D., Department of Otolaryngology, Head and Neck Surgery, Affiliated Sixth People's Hospital, Shanghai Jiao Tong University, Shanghai 200230, China. Email：021china@sina.com

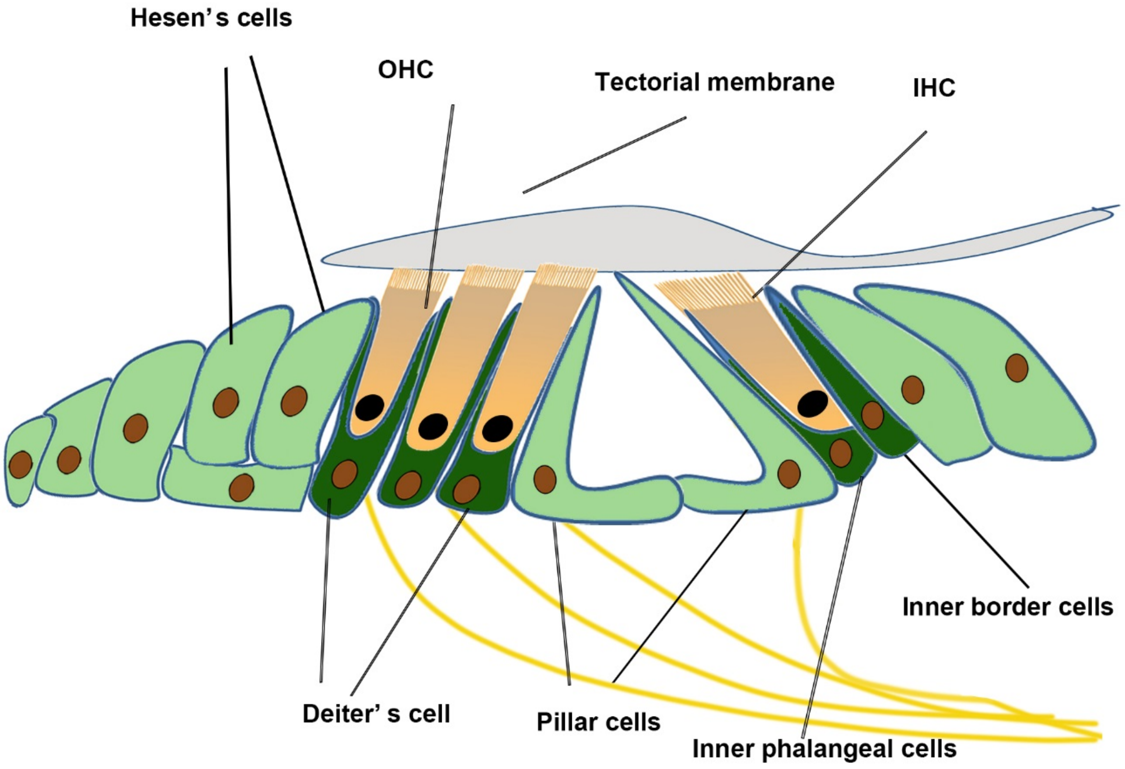

Supplementary Figure 1. A schematic of cochlear. The basilar membrane of the organ of Corti consists of the HCs and SCs. The mechanical motion of the epithelium was transducted into neural impluses by HCs. SCs include pillar cells, Deiter’s cells, inner border cells, Hensen’s cells, and inner phalangeal cells.

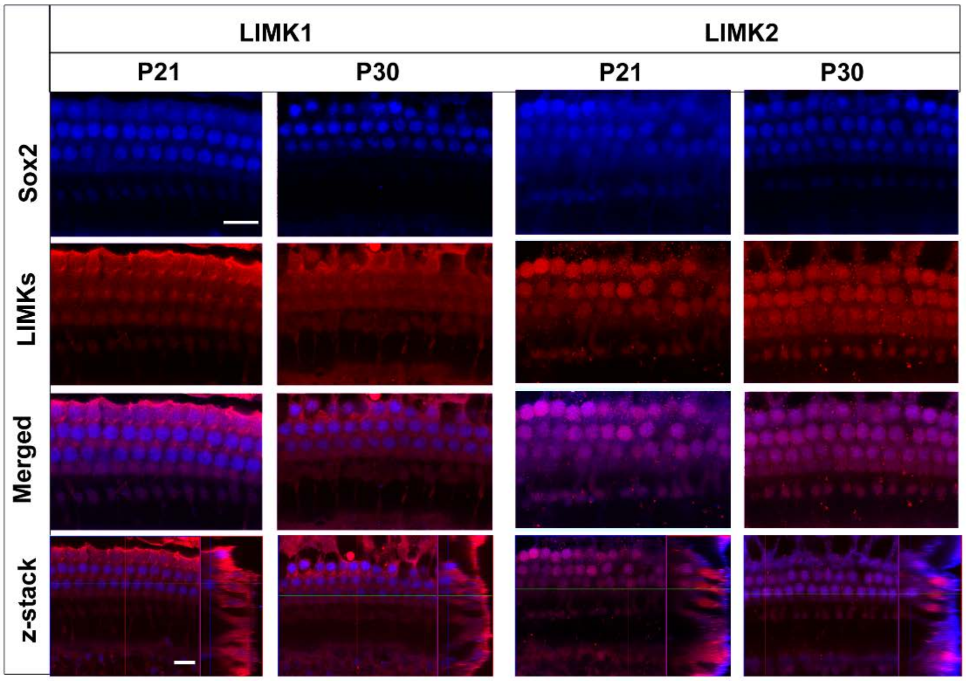

Supplementary Figure 2. Expression of LIMKs in the P21 and P30 WT mouse cochlea. Immunofluorescence staining showed that LIMK1 and LIMK2 were expressed in Deiter's cells and pillar cells. Sox2 was used as a marker for SCs. All images were taken from the apical and basal turns of the sensory epithelium. Scale bar = 10 μm.

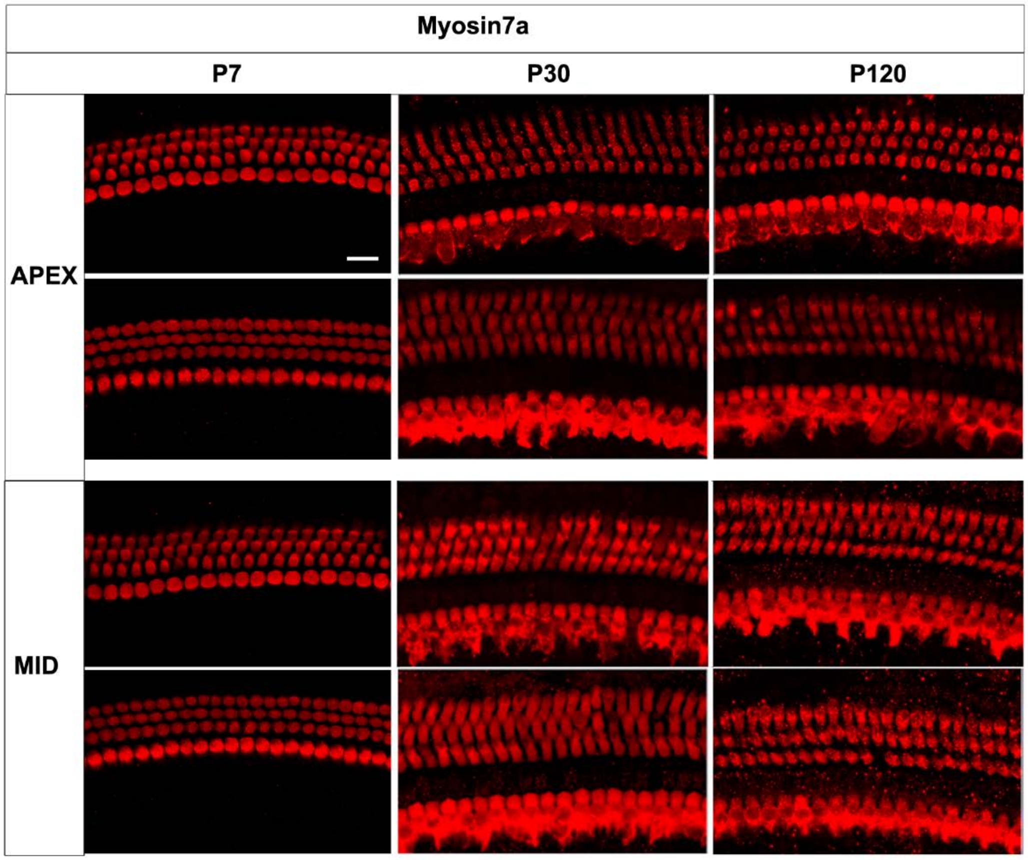

Supplementary Figure 3. The auditory HCs are morphologically normal in the DKO mice. Auditory HCs of P7, P30 and P120 mice were stained with antibodies against myosin7a and imaged using a confocal microscope. Images were taken from the apical and middle turns of the cochlea. Scale bar = 10 µm.

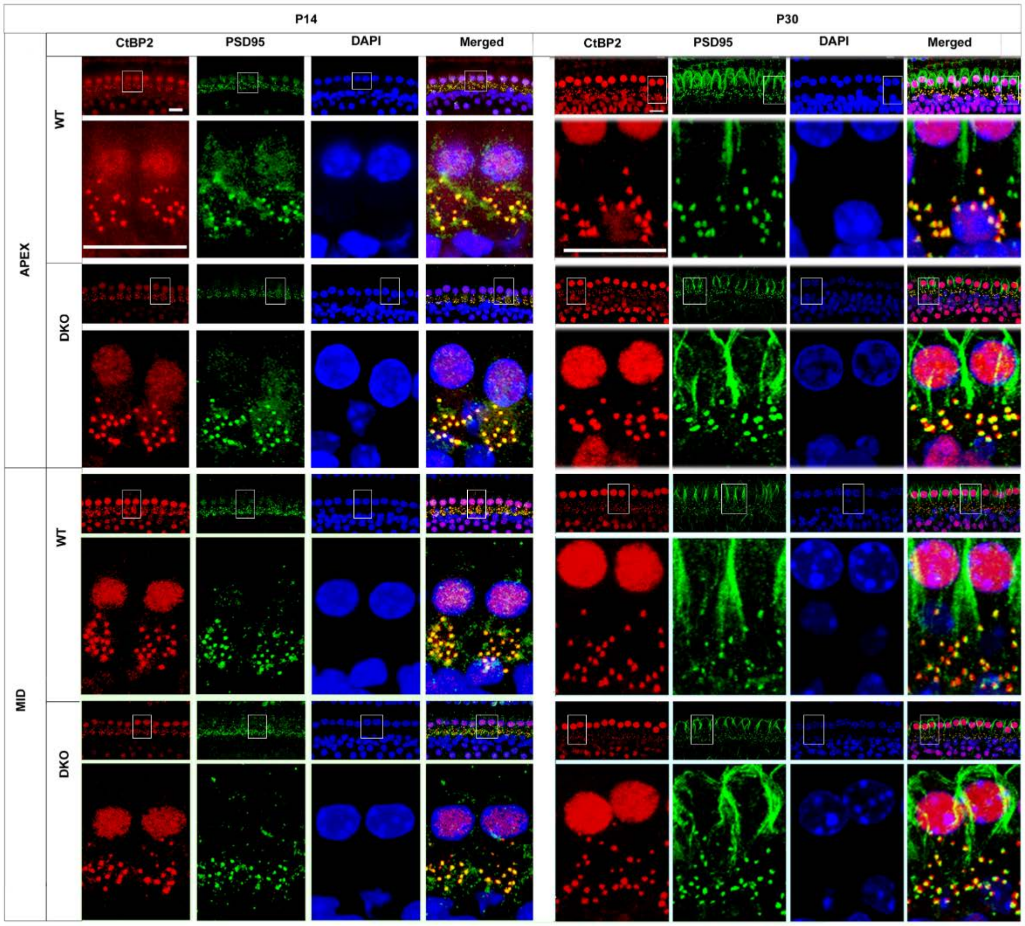

Supplementary Figure 4. The ribbon synapses were normal in DKO mice. (a) Ribbon synapses of P14 DKO and WT mice were stained with the ribbon synapse-specific markers CtBP2 and PSD95 and imaged under a confocal microscope. (b) Ribbon synapses of P30 DKO and WT mice were stained with the ribbon synapse-specific markers CtBP2 and PSD95 and imaged under a confocal microscope. Images were taken from the apical and middle turns of the cochlea. Scale bar = 10 µm.

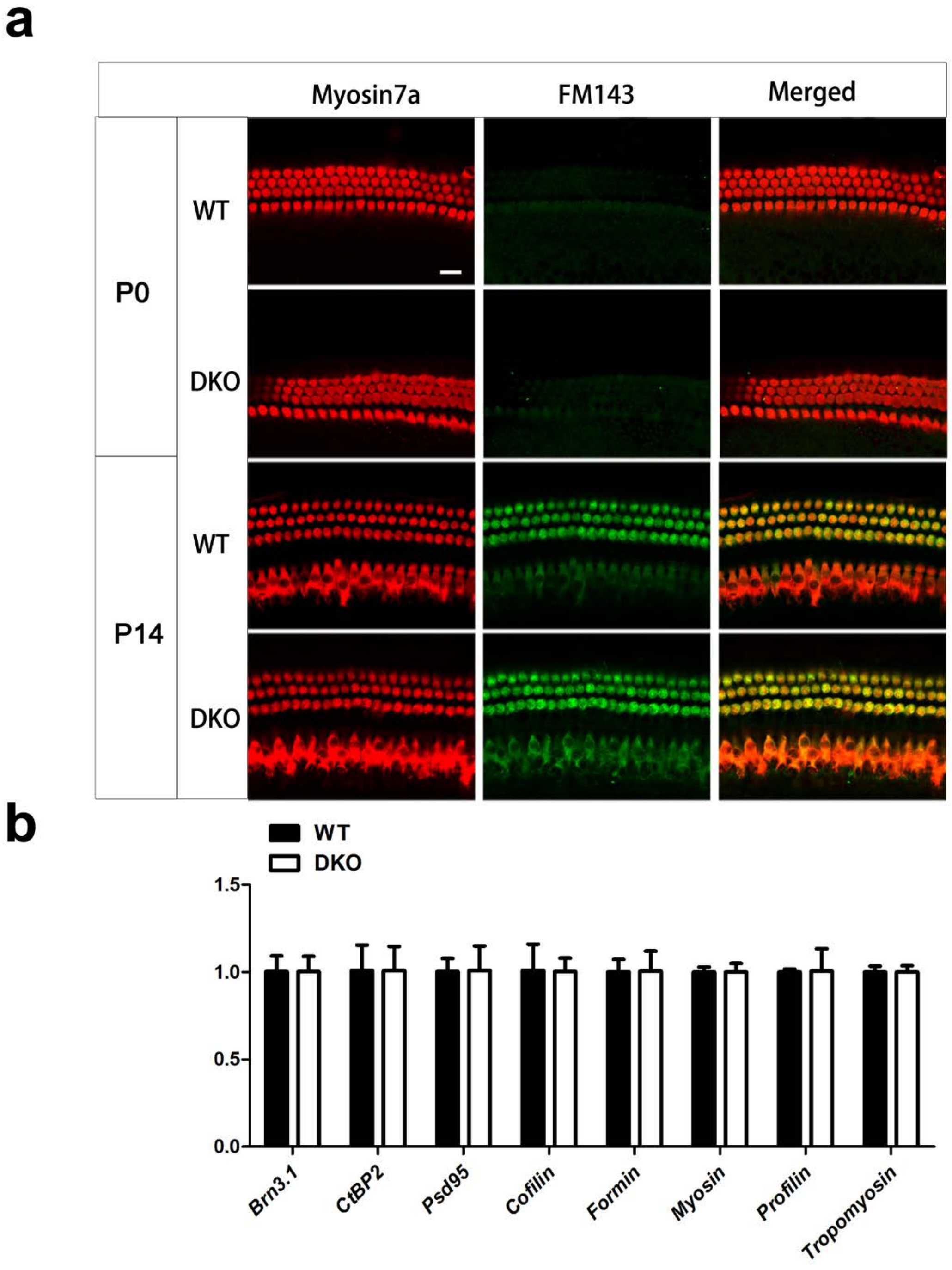
a
Myosin7a
FM143
Merged
P0
WT
DKO
P14
WT
DKO
b
WT
DKO
1.5
1.0
0.5
0.0
Brn3.1
CtBP2
Psd95
Cofilin
Formin
Myosin
Profilin
Tropomyosin

Supplementary Figure 5. The MET channel of OHCs and ribbon synapses are normal in DKO mice. (a) MET channels were normal in DKO mice. Auditory HCs of P0 and P14 mice were stained with FM1-43 dye and myosin7a antibody. Images were taken from middle turns of sensory epithelium. There was no difference in the immunolabeling signals from the apical to basal turns. Scale bar = 10 µm. (b) RT- qPCR was performed with P30 WT and DKO mice cochleae ($p > 0.05$, n = 5). Data are presented as means ± SD.
